# Physicians' attitude to allergen immunotherapy (AIT) prescription: an Italian survey

**DOI:** 10.1186/1939-4551-8-S1-A36

**Published:** 2015-04-08

**Authors:** Ambra Modiano

**Affiliations:** 1 Modena District, Specialized Assistance Department. Italy

## Background

Allergen Immunotherapy (AIT) is a valid treatment for respiratory allergy. Its effectiveness strictly relates to physicians’ attitude towards prescription and towards implemented strategies to favour adherence. We conducted a questionnaire-based survey among doctors usually prescribing AIT, to evaluate their approach to AIT prescription in routine clinical practice.

## Methods

A sample of 36 doctors (54 years, range 32-71; 12 males) working in health public setting (23) or private office (13) filled in an ad-hoc questionnaire. The items included: available time to visit patient and prescribe AIT; patient’s education to AIT or therapeutic alternatives; time dedicated to AIT description; barriers to AIT prescription; patients elective for AIT and contraindications; approach to poly-sensitized; explanation of advantages and drawbacks of different administration routes; use of informed consent.

## Results

Most doctors (58%) have 30’ for diagnosis and treatment prescription. Patients are always informed about the AIT existence. The majority of doctors reluctant to prescribe AIT, are discouraged by fear of adverse reactions (2%). Time spent to AIT description is 10’ in 72% of prescribers. All doctors quote the main differences of action between AIT and drugs. Most doctors follow guidelines for indication and contraindications and prescribe AIT to poly-sensitized patients (77%), with a maximum of two simultaneous desensitisations. In choosing sublingual or injections, patient’s opinion is regarded by 58% of doctors. Advantages and burdens of AIT are always exhibited, often distinguishing between the two routes (97%). Informed consent is required by 53% of doctors, 42% only for injections and 11% for both routes. Most doctors (69%) consider a problem prescribing AIT to immigrants.

## Conclusions

Despite the short available time, interviewed doctors appear very informative on AIT features during prescription. Patient’s values appear highly considered.

